# Electrical behavior and positive temperature coefficient effect of graphene/polyvinylidene fluoride composites containing silver nanowires

**DOI:** 10.1186/1556-276X-9-375

**Published:** 2014-08-01

**Authors:** Linxiang He, Sie-Chin Tjong

**Affiliations:** 1 Department of Physics and Materials Science, City University of Hong Kong, Hong Kong, China; 2 Shenzhen Research Institute, City University of Hong Kong, Hong Kong, China

**Keywords:** Graphene, Silver nanowires, Positive temperature coefficient, Polymer composite, Conductivity, Polyvinylidene fluoride

## Abstract

Polyvinylidene fluoride (PVDF) composites filled with in situ thermally reduced graphene oxide (TRG) and silver nanowire (AgNW) were prepared using solution mixing followed by coagulation and thermal hot pressing. Binary TRG/PVDF nanocomposites exhibited small percolation threshold of 0.12 vol % and low electrical conductivity of approximately 10^-7^ S/cm. Hybridization of TRGs with AgNWs led to a significant improvement in electrical conductivity due to their synergistic effect in conductivity. The bulk conductivity of hybrids was higher than a combined total conductivity of TRG/PVDF and AgNW/PVDF composites at the same filler loading. Furthermore, the resistivity of hybrid composites increased with increasing temperature, giving rise to a positive temperature coefficient (PTC) effect at the melting temperature of PVDF. The 0.04 vol % TRG/1 vol % AgNW/PVDF hybrid exhibited pronounced PTC behavior, rendering this composite an attractive material for making current limiting devices and temperature sensors.

## Background

Polymers with low weight, low production cost, and good corrosion resistance are favorable materials for making adhesives, membranes, circuit boards, electronic devices, etc. [1]. Most polymers are insulators with poor electrical conductivity. Their electrical conductivity can be improved markedly by adding large volume fractions of conductive metal particles and carbon blacks of micrometer dimensions. Polymer composites with large microfiller loadings generally exhibit poor processability and inferior mechanical strength [2-6]. In this regard, nanomaterials can be used as effective fillers for nanocomposite fabrication and property enhancements [7-9]. In particular, electrical properties of polymers can be enhanced greatly by adding low loading levels of graphene with high mechanical strength and electrical conductivity, forming conductive nanocomposites of functional properties [10,11]. Such nanocomposites have emerged as a promising and important class of materials for the electronics industry.

Graphene is a two-dimensional, monolayer sp^2^-bonded carbon with remarkable physical and mechanical properties. It has a large aspect ratio, exceptionally high mechanical strength, and excellent electrical and thermal conductivity [12]. Graphene fillers are generally prepared by oxidizing graphite flakes in strong acids to generate graphene oxide (GO). GO sheets are heavily oxygenated, bearing hydroxyl and epoxide functional groups on their basal planes, in addition to carbonyl and carboxyl groups at the sheet edges [13]. As a result, GO sheets can mix intimately with many organic polymers, facilitating the synthesis of GO/polymer composites with homogeneous dispersion of nanofillers. However, GO is an electrically nonconductive material; thus, the oxygenated functional groups must be removed either by reacting with chemical agents to form chemically reduced graphene or heating in a furnace to yield thermally reduced graphene (TRG) [10,11,14,15].

In a previous study, we reported the preparation of electrically conductive TRG/polymer composite by mixing GO in a polymer solution followed by hot pressing [16]. The in situ TRG sheets were dispersed homogenously in the polymer matrix. The main disadvantage of this approach, however, is that the percolated composites have a relatively low electrical conductivity, resulting from incomplete thermal reduction of GO. The low conductivity can greatly limit potential applications of the composites. In the past decade, the synthesis of one-dimensional metal nanomaterials has received great attention from chemists, materials scientists, and physicists. These materials include Ag [17,18], Cu [19,20], Au [21,22], and CuNi [23] nanowires (NWs). The incorporation of those nanowires with unique properties into polymers can yield novel composites with functional characteristics. For example, da Silva et al. incorporated CuNWs into polyvinylidene fluoride (PVDF) and found that the CuNW/PVDF nanocomposites exhibit high dielectric permittivity and low dielectric loss [24].

Conductive polymer composites generally show a large increase in electrical resistivity by heating near the glass transition or melting temperature of the polymer matrix. This behavior is widely known as the ‘positive temperature coefficient’ (PTC) effect. The mechanisms responsible for the PTC effect are rather complex. PTC effect may arise from a difference in thermal expansion coefficient between the polymer matrix and conductive fillers. In addition, other factors such as the type, size and dispersion state of fillers, and the type of polymers can affect the PTC behavior [25-38]. From the literature, many researchers have extensively studied the PTC behavior of polymer composites filled with carbon blacks (CBs) in the past two decades [26-32]. Kim et al. incorporated 40 to 60 wt % CBs (0.86 and 0.3 μm) into PVDF, polyacetal, polyester, polyamide-11, and polyamide-12 [29]. They reported that the PTC intensity of polymer composites was proportional to the polymer crystallinity. For example, the degree of crystallinity of the 50 wt % CB/polyamide-12 and 50 wt % CB/polyester composites was 48.30 and 36.26%, respectively. Thus, the former composite exhibited higher while the latter showed lower PTC intensity. Similarly, the 55 wt % CB (90 nm)/high-density polyethylene (HDPE) composite with large crystallinity exhibited higher PTC intensity than polypropylene (PP) composite at the same filler loading [30]. Recently, Dang et al. reported that the PP and HDPE composites with hybrid fillers of CBs (50 nm) and carbon fibers at 8 vol % loading exhibit strong PTC intensity [32]. They attributed this to the ease of a conducting network formation in the polymer matrix because of the large aspect ratio of carbon fibers. Analogously, hybridization of CBs (24 nm) with multiwalled carbon nanotubes also led to enhanced PTC intensity and reproducibility [31]. In this study, we aimed to improve electrical conduction behavior of TRG/PVDF composites by incorporating AgNWs. The AgNW/TRG/PVDF hybrid composites displayed interesting temperature-dependent electrical properties. PVDF is a semicrystalline polymer with high thermal stability, excellent chemical resistance, and high piezoelectric property.

## Methods

### Materials

Graphite flakes, ethylene glycol (EG), *N*,*N*-dimethylformamide (DMF), ferrite chloride (FeCl_3_), and poly (vinylpyrrolidone) (PVP) were purchased from Sigma-Aldrich (St. Louis, MO, USA). PVDF (Kynar 500) pellets were purchased from Arkema Inc. (King of Prussia, PA, USA). Silver nitrate (AgNO_3_) was obtained from Shanghai Chemical Reagent Company (Shanghai, China). All chemicals were used as received without further purification.

### Synthesis

**Figure 1:**
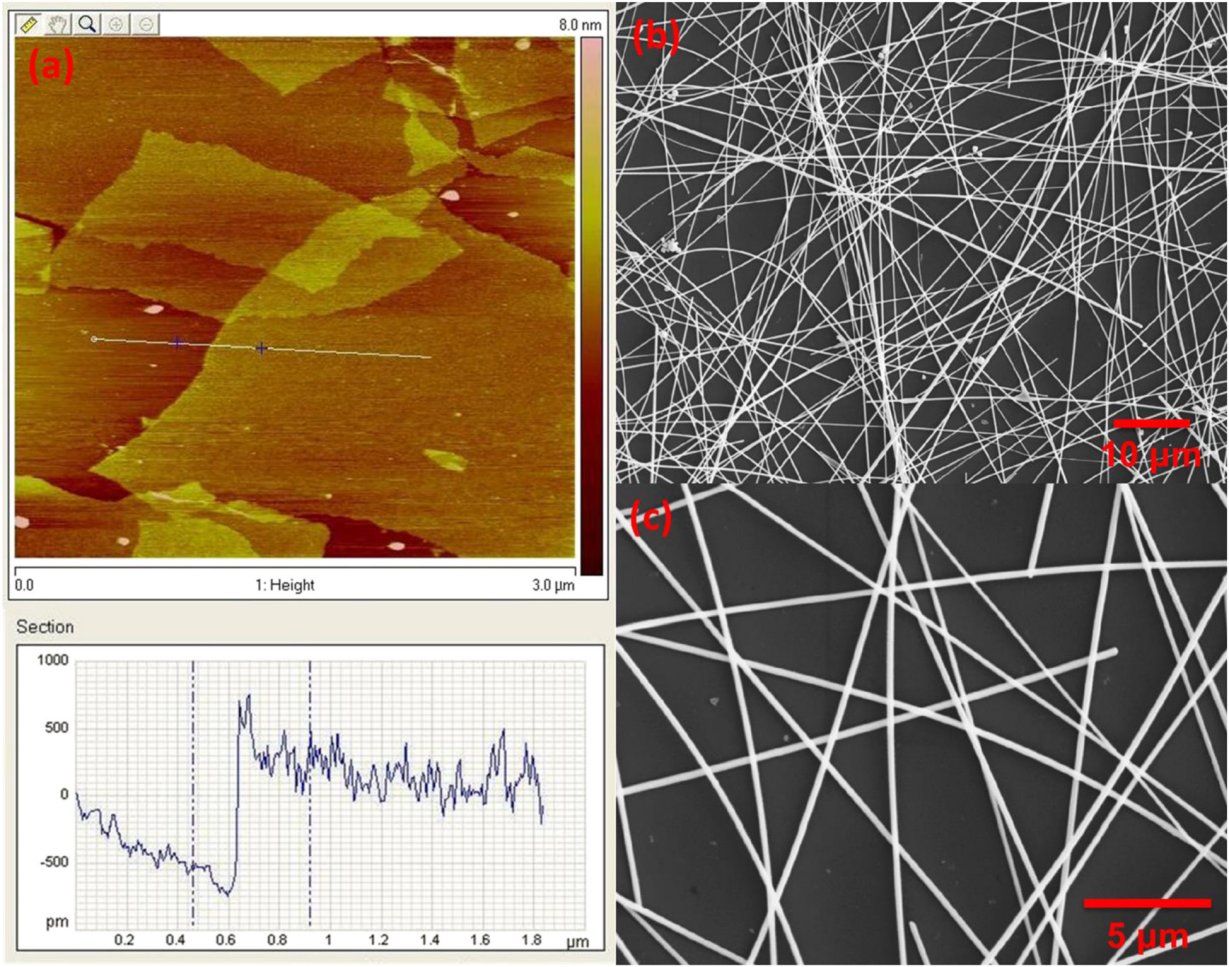
**AFM image of GO sheets and SEM micrographs of AgNWs. (a)** AFM image of GO sheets deposited onto a mica substrate. The line profile across GO shows a sheet thickness of approximately 1 nm. **(b, c)** SEM micrographs of the as-synthesized AgNWs at low and high magnifications.

Graphite oxide was prepared using a typical Hummers process [39] and can be readily exfoliated into monolayer GO sheets as displayed by atomic force microscopic (AFM) image (Figure 1a). The GO sheets were dispersed in DMF to generate a 2 mg/mL solution. AgNWs were synthesized according to the polyol method [18]. Typically, PVP (0.2 g) and AgNO_3_ (0.2 g) were dissolved in 20 ml EG at room temperature. Then, 60 μL of 0.5 mM FeCl_3_ solution (in EG) was pipetted, and the solution mixture was magnetically stirred for 5 min. Subsequently, the solution container was placed in an oil bath of 130°C and held at this temperature for 12 h. The obtained AgNW products were washed with ethanol for five times and then re-dispersed in DMF. The average diameter and length of nanowires were approximately 130 nm and 110 μm, respectively (Figure 1b,c), producing an average aspect ratio of approximately 850.

The TRG/PVDF composites were prepared based on our previous strategy [16]. To prepare AgNW/TRG/PVDF composites, the PVDF solution (in DMF), GO solution (in DMF), and AgNW dispersion (in DMF) with appropriate mass ratios were mixed under sonication for 20 min. The mixed suspension was then coagulated into a large amount of stirring water. The precipitated fibrous mixture was washed with distilled water and ethanol and then collected using vacuum filtration. By drying at 70°C overnight, the fibrous mixture was finally hot-pressed at 200°C. This process converted GO to TRG [15], thereby forming AgNW/TRG/PVDF hybrid composites. The composite samples were pressed into sheets of about 0.5 mm thick for the electrical characterization.

### Characterization

The morphology of AgNWs and AgNW/TRG/PVDF composites were examined in scanning electron microscopes (SEMs; JEOL JSM 820 and JEOL FEG JSM 6335; JEOL Ltd., Akishima-shi, Japan). Static electrical conductivity of the composites was measured with an Agilent 4284A Precision LCR Meter (Agilent Technologies, Inc., Santa Clara, CA, USA). The specimen surfaces were coated with silver ink to form electrodes. Moreover, the specimens were placed inside a computer-controlled temperature chamber to allow temperature-dependent conductivity measurements.

## Results and discussion

**Figure 2:**
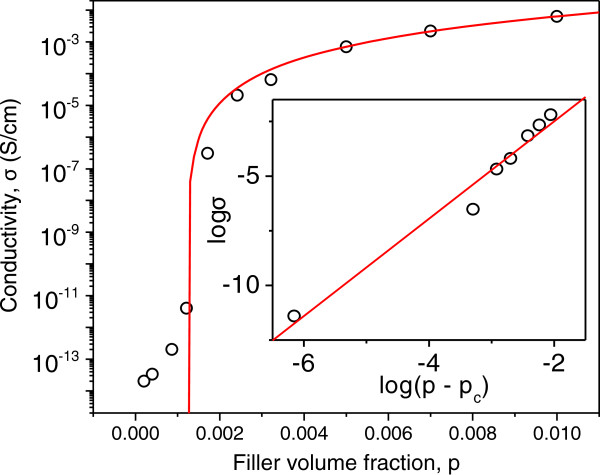
**Electrical conductivity of TRG/PVDF composites as a function of TRG content.** Inset, log *σ* vs. log(*p* – *p*_c_) plot. Close circles are data points. Red solid lines in both graphs are calculated conductivities by fitting experimental data to Equation 1. Fitting results are *p*_c_ = 0.12 ± 0.02 vol %, *t* = 2.61 ± 0.22, and *σ*_0_ = 1,496.43 ± 136.38 S/cm.

Figure 2 shows static electrical conductivity of the TRG/PVDF composites at room temperature. From the percolation theory, a rapid increase in electrical conductivity occurs when the conductive fillers form a conductive path across the polymer matrix of a composite. The conductivity of the composite *σ*(*p*) above the percolation threshold (*p*_c_) is given by [40,41]:

(1) $$\sigma \left(p\right)\;=\sigma_{0}{\left(p–p_{c}\right)}^{t},\mathrm{for}\;p>p_{c}$$

where *p* is the filler content and *t* the critical exponent. Nonlinear fitting in Figure 2 gives *p*_c_ = 0.12 vol %. We attribute the low *p*_c_ to the high aspect ratio of TRG sheets, which lead to easier connectivity in forming a conductive network. Although the TRG/PVDF composites have a small *p*_c_, their conductivity at *p*_c_ is quite low, i.e., in the order of approximately 10^-7^ S/cm. Such a low conductivity renders percolating TRG/PVDF composites can be used only for antistatic applications. From Figure 2, the conductivity reaches approximately 5 × 10^-3^ S/cm at 1 vol % TRG. As recognized, TRGs still contain residual oxygenated groups despite high temperature annealing [15]. In other words, TRGs are less conductive than pristine graphene. To improve electrical conductive properties, AgNWs are added to the TRG/PVDF composites as hybridized fillers.

**Figure 3:**
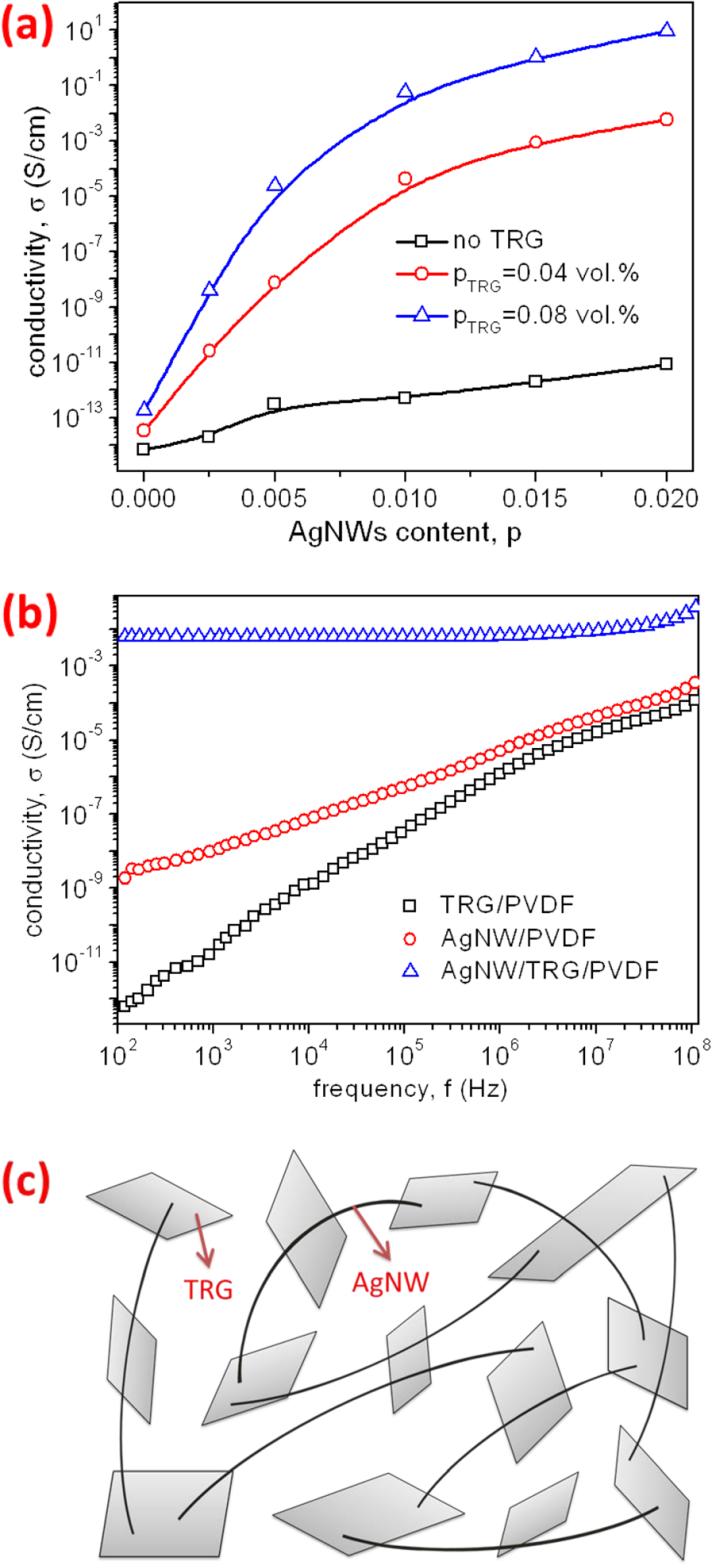
**Effect of AgNW content, AC conductivity, and schematic diagram of hybrid composite. (a)** Effect of AgNW content on electrical conductivity of AgNW/TRG/PVDF hybrid composites. **(b)** AC conductivity of 0.04 vol % TRG/PVDF, 2 vol % AgNW/PVDF, and 2 vol % AgNW/0.04 vol % TRG/PVDF composites. **(c)** Schematic diagram of hybrid composite filled with AgNWs and TRGs. Filler hybridization facilitates the formation of a conducting network.

**Figure 4:**
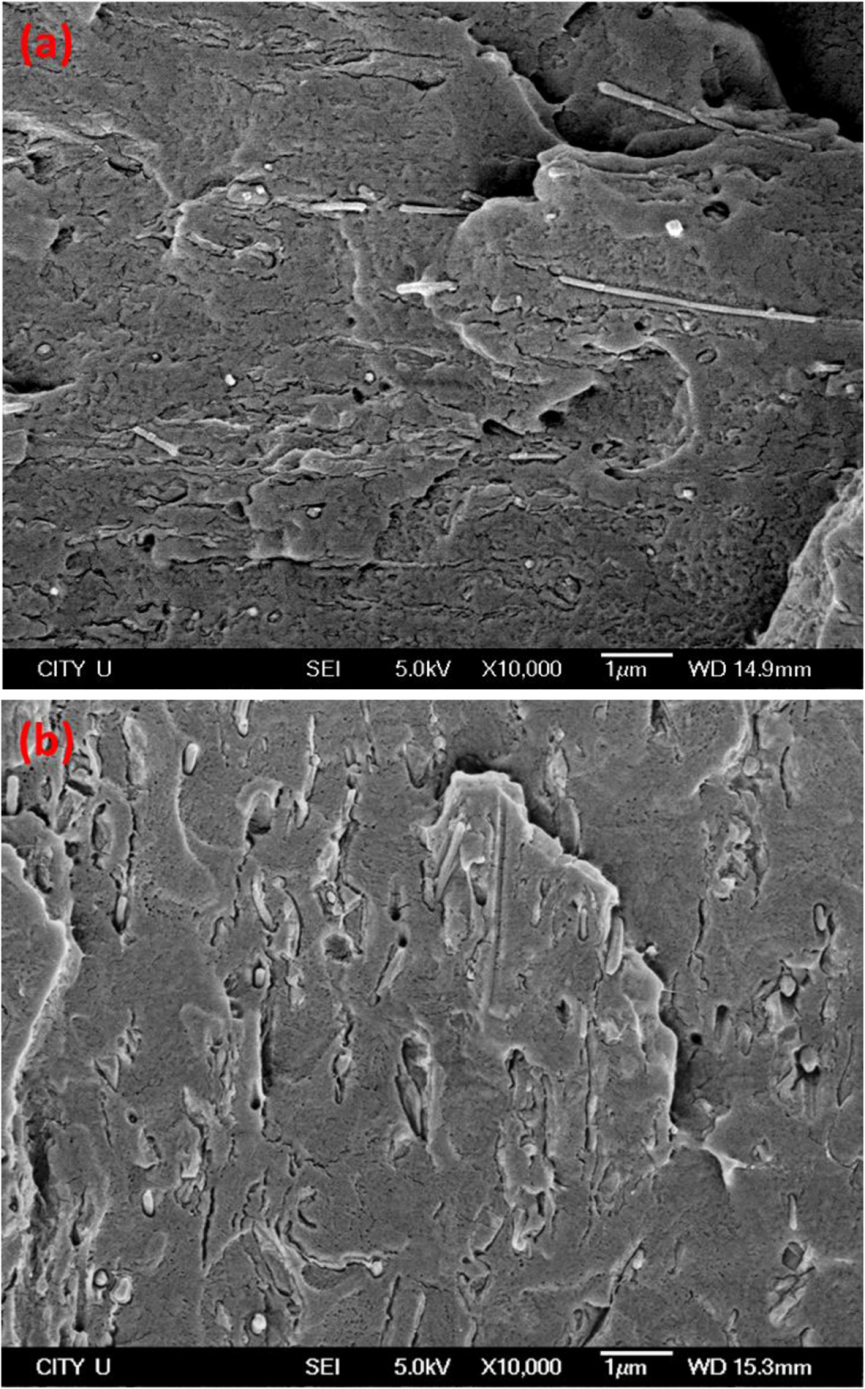
**SEM micrographs of hybrid composites.** SEM micrographs of AgNW/TRG/PVDF composites with **(a)***p*_AgNW_ = 0.5 vol % and *p*_TRG_ = 0.04 vol % and **(b)***p*_AgNW_ = 1 vol % and *p*_TRG_ = 0.04 vol %.

**Figure 5:**
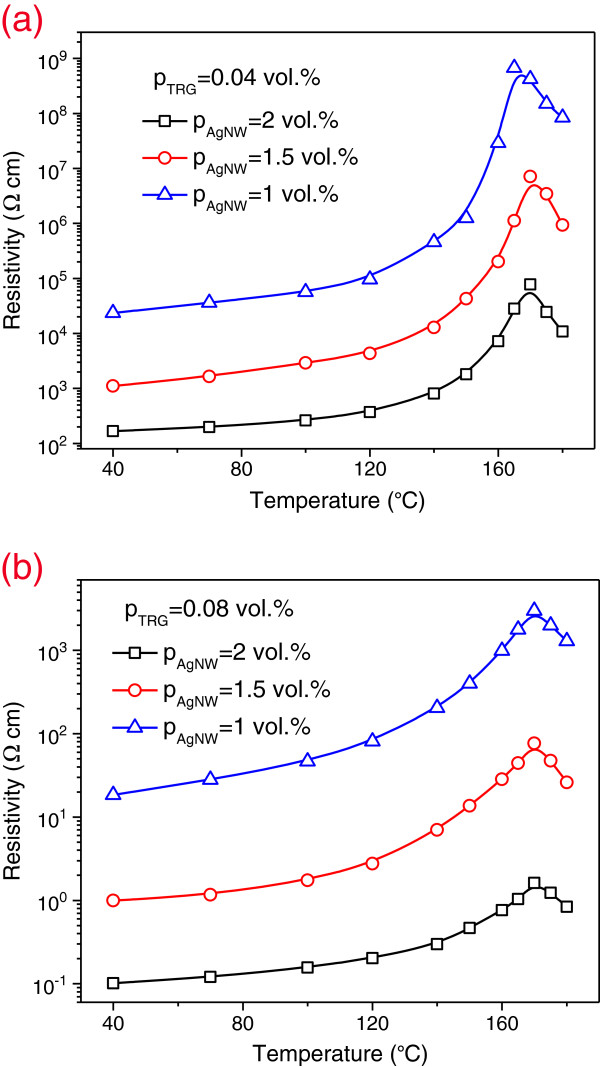
**Effect of temperature on resistivity of AgNW/TRG/PVDF composites with (a) *p***_**TRG**_ **= 0.04 vol % and (b) *p***_**TRG**_ **= 0.08 vol %.**

Figure 3a shows the effect of AgNW addition on electrical conductivity of AgNW/TRG/PVDF hybrids. Apparently, electrical conductivity of the 0.04 vol % TRG/PVDF and 0.08 vol % TRG/PVDF composites increases with increasing AgNW content, especially for latter hybrid composite system. This is mainly due to the very high conductivity of AgNWs (6.3 × 10^5^ S/cm) and the creation of new electrical contacts by nanowires. In the case of AgNWs alone, the AgNW/PVDF composites show no percolation up to 2 vol % filler loading. By adding small amounts of TRGs (0.04 and 0.08 vol %), the hybrids display a steady increase in conductivity with increasing Ag content. Interestingly, the conductivity of AgNW/TRG/PVDF hybrids is much higher than the total conductivity of both TRG/PVDF and AgNW/PVDF composites. Thus, there exists a synergetic effect between these two types of nanofillers [42]. It seems that AgNWs can bridge the TRG sheets effectively, facilitating the transport of electrons among them [43]. The presence of conducting network can be detected by the alternating current (AC) response that manifested itself in a conductivity plateau. Figure 3b shows the AC conductivity of PVDF filled with TRGs, AgNWs, and hybrid nanofillers. For the TRG/PVDF and AgNW/PVDF composites, electrical conductivity rises almost linearly with the frequency, implying these materials are insulators. In contrast, the conductivity of AgNW/TRG/PVDF composite is frequency independent from 10^2^ to 10^7^ Hz. This sample exhibits a DC conductivity plateau over a broad frequency range, showing the formation of good conducting network. Figure 3c is a schematic diagram illustrating the occurrence of synergistic effect between the AgNW and TRG fillers in a conductive network. On the contrary, the AgNW or TRG filler alone does not form a conducting path. The percolated AgNW/TRG/PVDF composite exhibits higher conductivity compared to a combined total conductivity of TRG/PVDF and AgNW/PVDF composites. From Figure 3a, the conductivity of 1 vol % AgNW/0.04 vol % TRG/PVDF hybrid is more than nine orders of magnitude higher than that of the 1 vol % AgNW/PVDF composite. Furthermore, the conductivity of 2 vol % AgNW/0.08 vol % TRG/PVDF, i.e., 10 S/cm is comparable to that of measured graphite paper with a conductivity of 12 S/cm [44]. Figure 4a,b is the SEM micrographs showing typical morphologies of hybrid composites. The AgNWs are well dispersed within the polymer matrix. The use of sonication during the composite fabrication process can reduce the aspect ratio of AgNWs as expected.The effect of temperature (40 to 180°C) on electrical resistivity (a reciprocal of conductivity) of AgNW/TRG/PVDF hybrids is now discussed (Figure 5). All hybrid composites show a slow increase in resistivity with increasing temperature initially followed by a sharp increase in resistivity as the temperature approaches melting point of PVDF. This behavior is commonly referred to as the positive temperature coefficient (PTC) effect of resistivity. A maximum increase in resistivity is particularly apparent for the composite with 0.04 vol % TRG and 1 vol % AgNW loadings, being more than four orders of magnitude higher than that at 40°C. Above the melting temperature of PVDF, a reverse effect, i.e., a decrease in resistivity or negative temperature coefficient (NTC) can be seen. This decrease is due to the re-aggregation of conductive fillers in molten polymer, generating a conductive path in the composite. It is observed that the hybrids with higher AgNW content exhibit weaker PTC effect, demonstrating that their conductive network is more robust than those with lower AgNW content. By utilizing AgNWs as a hybrid filler component, we can tune the PTC intensity in electrically conductive TRG/polymer composites effectively.

Recently, Ansari and Giannelis prepared TRGs by fast heating GOs in a furnace at 1,000°C for 30 s [36]. The PTC effect was not found in solution-mixed 3 to 4 wt % TRG/PVDF nanocomposites. Instead, the resistivity of such nanocomposites decreased from ambient to 170°C, displaying NTC effect behavior. They attributed this to the higher aspect ratio of TRGs such that the contact resistance dominated over tunneling resistance. More recently, Rybak et al. studied electrical conducting behavior of HDPE and polybutylene terephthalate (PBT) filled with Ag spherical nanoparticles (150 nm) [38]. The percolation threshold of Ag/HDPE and Ag/PBT nanocomposites was determined to be 17.4 and 13.8 vol %, respectively. Silver spherical nanoparticles exhibited low aspect ratio of unity, leading to large percolation threshold of these nanocomposites as expected. Furthermore, percolated Ag/HDPE and Ag/PBT nanocomposites also displayed PTC characteristics. Comparing with binary Ag/HDPE and Ag/PBT composites, our ternary hybrid composites only require very low AgNW additions, i.e., 1 to 2 vol % to achieve the PTC effect. Such low AgNW additions are beneficial for industrial applications, because AgNWs with high aspect ratio are more cost-effective than Ag nanoparticles of large volume fractions.

**Figure 6:**
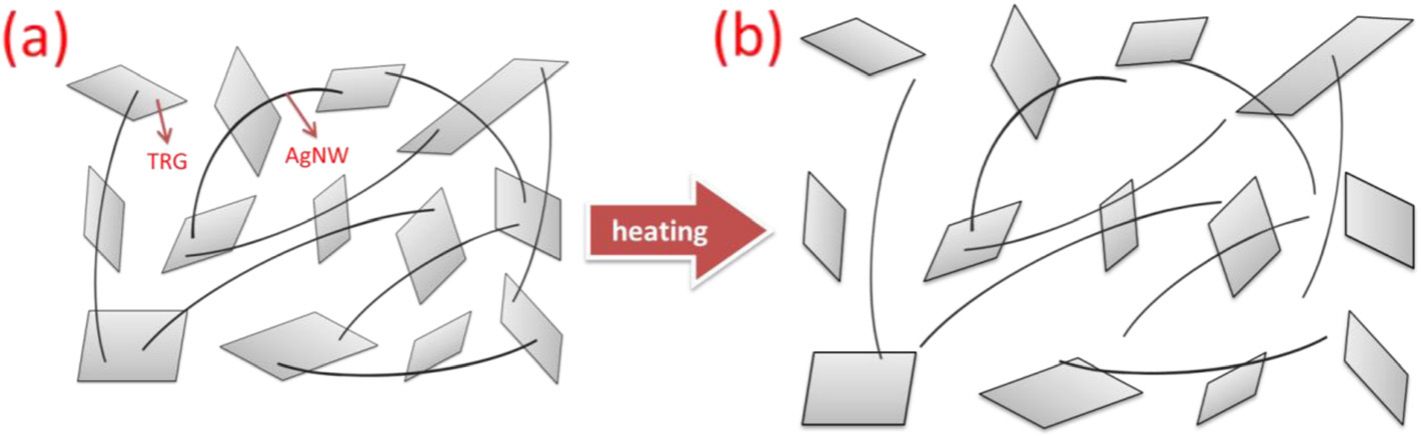
Schematic diagrams showing the dispersion of TRGs and AgNWs in a hybrid (a) before and (b) after heating.

For electrically conductive polymer composites, two types of resistance can develop normally: constriction contact resistance and tunneling contact resistance [36]. At low filler loadings, the fillers are dispersed at a large distance so that a conducting network cannot form in insulating polymer matrix. Under such a circumstance, electrical conduction occurs due to the ‘Zener tunneling or internal field emission effect,’ i.e., electrons penetrate a potential barrier because of magnified local electrical field [45,46]. Generally, the number of contacts increases with an increase in the number of filler particles of large aspect ratio, so the contact resistance predominates. In this case, the filler particles link one another to form a conducting network throughout the system, leading to high conductivity of the composite. As recognized, molecular chain movement is activated when the temperature exceeds glass transition temperature of the polymer. For the AgNW/TRG/PVDF composite, TRGs can make many contacts with the polymer matrix because of their large surface-to-volume ratio. Thus, low-density TRGs sense quickly to the movement of polymer molecular chains as the temperature increases. In contrast, AgNWs with higher density respond slowly to molecular chain movement. An increase in temperature can disrupt conductive path network by increasing the distance between TRG fillers as shown in Figure 6a,b. The separation of AgNWs and TRGs due to heating causes a reduction in the overall contacts among AgNWs and TRGs, resulting in a gradual increase in resistivity. PTC materials generally find useful applications for fabricating temperature sensors and self-regulating or current limiting devices [47,48]. The pronounced PTC behavior of the AgNW/TRG/PVDF composites enables the materials to respond very rapidly to the changes in temperature. Thus, the hybrids are novel PTC materials finding attractive usage in industrial sectors for a variety of smart and functional applications.

## Conclusions

AgNW/TRG/PVDF hybrid composites were prepared using solution mixing followed by coagulation and thermal hot pressing. Electrical measurements showed that the bulk conductivity of hybrids was higher than a combined total conductivity of both TRG/PVDF and AgNW/PVDF composites at the same filler loading. This was due to the AgNWs bridged TRG sheets effectively in forming a conductive network in the PVDF matrix, producing a synergistic effect in conductivity. Consequently, electrical conductivity of 2 vol % AgNW/0.08 vol % TRG/PVDF composite was comparable to measured conductivity of graphite paper. Finally, the resistivity of hybrid composites increased with increasing temperature, particularly at the melting temperature of PVDF, generating a pronounced PTC effect. This effect was caused by the volume expansion of PVDF matrix with increasing temperature, which disrupted the synergistic effect and reduced electrical contacts among the conductive fillers.

## Competing interests

The authors declare that they have no competing interests.

## Authors’ contributions

LH carried out the experiments, interpreted the data, and drafted the manuscript. SCT participated in the design of the study, material analysis, and revision of the whole manuscript. Both authors read and approved the final manuscript.
